# SOUND HEALTH: HARNESSING MUSIC FOR HEALTH AND WELL-BEING IN SERVICE OF OLDER PEOPLE

**DOI:** 10.1093/geroni/igz038.112

**Published:** 2019-11-08

**Authors:** Emmeline Edwards

**Affiliations:** National Institutes of Health, National Center for Complementary and Integrative Health, Bethesda, Maryland, United States

## Abstract

Music can get you moving, lift your mood, help you recall a memory, and can potentially improve your health. A partnership between the National Institutes of Health and the John F. Kennedy Center for the Performing Arts, called Sound Health is expanding current knowledge and understanding of how listening, performing, or creating music involves intricate circuitry in the brain that could be harnessed for health and wellness. The presentation will focus on research that shows that music provides cognitive, socio-emotional, motor benefits as well as evidence for neural plasticity. The potential for music as therapy for several neurological disorders associated with aging will be discussed. Dr. Edwards will also highlight research gaps and opportunities in basic/mechanistic and clinical research on music and the aging brain.

